# *Schistosoma mansoni* infection induces hepatic metallothionein and S100 protein expression alongside metabolic dysfunction in hamsters

**DOI:** 10.1093/pnasnexus/pgae104

**Published:** 2024-03-07

**Authors:** Parviz Ghezellou, Verena von Bülow, David Luh, Elisa Badin, Wendell Albuquerque, Martin Roderfeld, Elke Roeb, Christoph G Grevelding, Bernhard Spengler

**Affiliations:** Institute of Inorganic and Analytical Chemistry, Justus Liebig University Giessen, 35392 Giessen, Germany; Department of Gastroenterology, Justus Liebig University Giessen, 35392 Giessen, Germany; Institute of Inorganic and Analytical Chemistry, Justus Liebig University Giessen, 35392 Giessen, Germany; Institute of Inorganic and Analytical Chemistry, Justus Liebig University Giessen, 35392 Giessen, Germany; Institute of Food Chemistry and Food Biotechnology, Justus Liebig University Giessen, 35392 Giessen, Germany; Department of Gastroenterology, Justus Liebig University Giessen, 35392 Giessen, Germany; Department of Gastroenterology, Justus Liebig University Giessen, 35392 Giessen, Germany; Institute of Parasitology, Biomedical Research Center Seltersberg (BFS), Justus Liebig University Giessen, 35392 Giessen, Germany; Institute of Inorganic and Analytical Chemistry, Justus Liebig University Giessen, 35392 Giessen, Germany

**Keywords:** schistosomiasis, proteomics, lipidomics, mass spectrometry imaging, hamster liver

## Abstract

Schistosomiasis, a widespread neglected tropical disease, presents a complex and multifaceted clinical-pathological profile. Using hamsters as final hosts, we dissected molecular events following *Schistosoma mansoni* infection in the liver—the organ most severely affected in schistosomiasis patients. Employing tandem mass tag–based proteomics, we studied alterations in the liver proteins in response to various infection modes and genders. We examined livers from female and male hamsters that were: noninfected (control), infected with either unisexual *S. mansoni* cercariae (single-sex) or both sexes (bisex). The infection induced up-regulation of proteins associated with immune response, cytoskeletal reorganization, and apoptotic signaling. Notably, *S. mansoni* egg deposition led to the down-regulation of liver factors linked to energy supply and metabolic processes. Gender-specific responses were observed, with male hamsters showing higher susceptibility, supported by more differentially expressed proteins than found in females. Of note, metallothionein-2 and S100a6 proteins exhibited substantial up-regulation in livers of both genders, suggesting their pivotal roles in the liver's injury response. Immunohistochemistry and real-time-qPCR confirmed strong up-regulation of metallothionein-2 expression in the cytoplasm and nucleus upon the infection. Similar findings were seen for S100a6, which localized around granulomas and portal tracts. We also observed perturbations in metabolic pathways, including down-regulation of enzymes involved in xenobiotic biotransformation, cellular energy metabolism, and lipid modulation. Furthermore, lipidomic analyses through liquid chromatography–tandem mass spectrometry and matrix-assisted laser desorption/ionization mass spectrometry imaging identified extensive alterations, notably in cardiolipin and triacylglycerols, suggesting specific roles of lipids during pathogenesis. These findings provide unprecedented insights into the hepatic response to *S. mansoni* infection, shedding light on the complexity of liver pathology in this disease.

**Significance Statement**

Schistosomiasis, caused, among others, by the parasite species *Schistosoma mansoni*, is a significant public health concern affecting millions of people worldwide. We investigated the huge impact of *S. mansoni* infection on liver proteome and lipidome in both genders of a rodent infection model. Using modern analytical and biochemical methods, we uncovered substantial alterations in protein expression and metabolic pathways. Among others, metallothionein-2 and S100a6 proteins showed marked elevations postinfection, underscoring their pivotal roles. Host gender-based differences in immune response were observed, with males exhibiting higher susceptibility. The infection-induced metabolic shifts, particularly in lipid metabolism, point to potential disruptions in mitochondrial function and energy production. These findings deepen our understanding of schistosomiasis pathology and offer insights into possible therapeutic interventions.

## Introduction

Schistosomiasis is a neglected tropical disease affecting more than 230 million people worldwide (1). The burden of the disease was estimated at 1.5 million disease-adjusted life years, which is of major public health concern associated with a substantial socioeconomic burden on patients and their households (2). Trematode parasites of the genus *Schistosoma* are responsible for the disease, besides other species mainly *Schistosoma haematobium*, *Schistosoma japonicum*, and *Schistosoma mansoni*. In *S. mansoni* infection, the adult male and female worms reside and mate in the veins of their mammalian host and produce >300 eggs daily. Some eggs become permanently trapped in the host's liver tissue and generate granulomatous lesions, which initiate inflammation, immune responses, and finally liver fibrosis (3). Other organs, such as gut, spleen, kidney, and lung, can be affected to varying degree by schistosomiasis, making it a disease of considerable complexity (4). Praziquantel (PZQ), a broad-spectrum anthelmintic, is the only commonly used drug to treat schistosomiasis. However, PZQ's widespread use justifies drug resistance development concerns (5, 6). Furthermore, endemic populations can reinfect when exposed to water with cercariae, the infectious stage of the life cycle of schistosomes, making one-time PZQ administration ineffective (7). Consequently, it is crucial to fully understand the mechanisms of pathology to help developing new treatments and reducing the disease burden in the face of the likely emergence of drug resistance (5).

With the rapid advancement of modern analytical techniques, molecular studies have provided in-depth information on parasite and host biology, which helps to understand the clinical/pathological processes occurring in the liver upon infection. In this context, mass spectrometry (MS)-based omics technologies have proven as powerful analytical methods for pathogen research and disease-associated pathological processes (8). So far, schistosome proteomics has mainly focused on parasite composition rather than host protein profile alterations during active schistosomiasis (5). However, several studies have shown that *S. mansoni* infection could alter the expression of hepatic proteins in murine models, suggesting that schistosomiasis significantly impacts distinct functions of target organs (9–11). We recently showed by different analytical methods, such as matrix-assisted laser desorption/ionization MS imaging (MALDI-MSI) and liquid chromatography–tandem MS (LC–MS/MS), that *S. mansoni* eggs change hepatic lipid and carbohydrate metabolism, which damages DNA in the host parenchyma due to oxidative stress (12, 13).

To investigate the abundance changes of hepatic proteins in response to *S. mansoni* infection in yet unprecedented detail, we employed high-resolution quantitative proteomics utilizing the isobaric label tandem mass tag (TMT) approach. We conducted a quantitative comparison of liver proteomes in hamsters (*Mesocricetus auratus*) infected with *S. mansoni* cercariae of both sexes (bisex; bs), single-sex (ss), and a noninfected (ni) control. Furthermore, we validated the alterations in the most strongly regulated proteins through quantitative real-time PCR (RT-qPCR) and immunohistochemistry. To gain a detailed insight into the metabolic consequences of these protein dysregulations, we additionally performed MALDI-MSI and LC-MS/MS lipidomic analyses of the livers of both bs and ni groups. Our findings uncovered profound alterations in the liver proteome and lipidome, highlighting the substantial influence of both the mode of infection (bisex vs. ss) and the gender of the animal model (female and male) on the host’s response to schistosome infection. Our results substantially extend previous knowledge of processes and molecules involved in the host–parasite interactions at the level of hepatic responses to schistosome infection.

## Results and discussion

### Study overview and the liver proteomics workflow

**Fig. 1.**
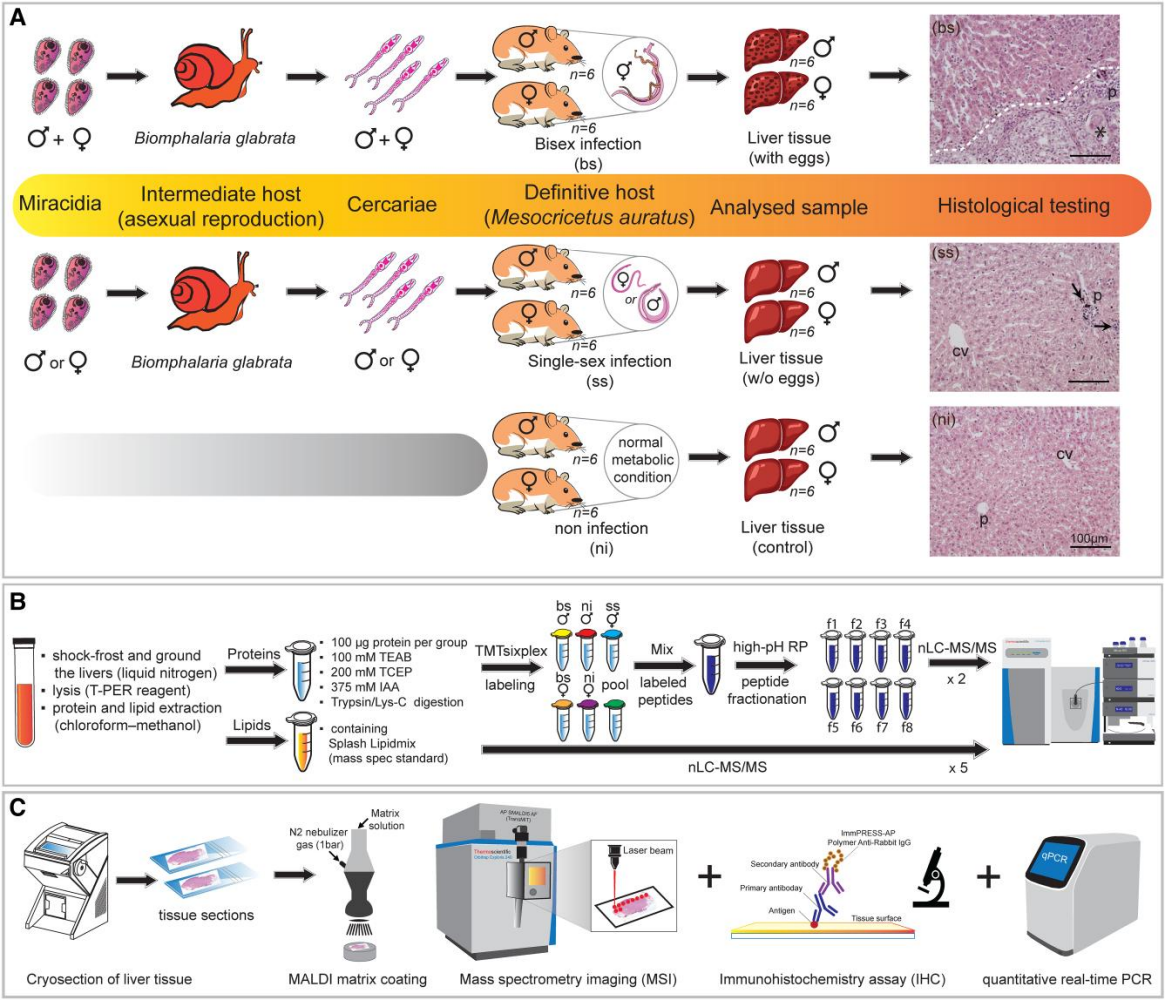
Schematic representation of the workflow used in this study. A) Syrian hamsters (*M. auratus*) were used as final hosts for *S. mansoni*, and snails (*B. glabrata*) as intermediate hosts. Female and male hamsters were separated into three main groups, including ni hamsters as controls (ni; *n* = 12), hamsters infected with only one sex of *S. mansoni* cercariae (ss; *n* = 12), and hamsters infected with both sexes of cercariae (bs; *n* = 12). Liver tissue of hamsters was harvested and used for the next steps. The liver sections were stained with H&E to visualize hepatic architecture. CV, central vein; PF, portal field; dashed line, border of granuloma; arrows, inflammatory infiltration; asterisk, *S. mansoni* egg. B) The hepatic proteome and lipidome were extracted and analyzed for quantitative assays using nano-LC coupled to high-resolution tandem mass spectrometry (nLC-HR-MS/MS). C) The liver sections were processed to apply MSI, IHC, and qPCR methods. The animation was created with Adobe Illustrator.

The previous experimental data from our groups have highlighted a range of processes that are influenced in the liver of hamsters upon infection with *S. mansoni*, such as hepatic inflammation, alterations in hepatic metabolism, and modulation of immune responses (12, 13). In these studies, we obtained first hints for infection-associated effects in the final host after bs and ss infection. Therefore, we speculated that the mode of infection (bs vs. ss) may differentially influence processes in the liver as one of the main target organs (besides gut and spleen) for schistosome eggs. Evidence in the literature also indicates that gender-related factors play a role in immune regulation, which in turn contributes to the development of fibrosis and the chronicity of infection (14–17). Therefore, we extended our approach, including female and male hamsters, to investigate a gender influence. In total, we analyzed the livers of 36 hamsters (*M. auratus*) to reveal alterations of the liver proteome and lipidome in response to *S. mansoni* infection (Fig. 1A). Of these, 12 hamsters were infected with *S. mansoni* cercariae of bs and 12 hamsters with ss infected, while 12 ni hamsters were used as control (ni; Fig. 1A). Compared with ni controls, hematoxylin and eosin (H&E) staining of hepatic tissue samples showed histological changes, e.g. portal inflammatory infiltrations in ss and egg-induced granulomas in bs hamsters (Figs. 1A and S1), indicating similar pathologic changes as observed in infected humans (3).

Livers were sorted into six biological groups (*n* = 6, hamsters/group) according to the gender of hamsters (male and female) and the infection mode (ni, ss, and bs) and used for further analysis (Fig. 1B and C). For proteomic analyses, samples were labeled with TMT 6-plex isobaric reagents, including one identical pooled sample for normalization (Fig. 1B). TMT-labeled peptides were combined and prefractionated offline into the eight main fractions using high-pH reversed-phase columns to decrease sample complexity in each fraction before nano-scale LC-MS/MS (nLC-MS/MS) analysis. Peptides were subsequently analyzed using a hybrid quadrupole—orbital trapping high-resolution mass analyzer, characterized by high mass accuracy, sensitivity, and sequencing speed, resulting in improved proteome depth and protein coverage (18, 19). We used a false discovery rate (FDR) of 1% on the peptide-spectrum match and protein levels for data analysis in MaxQuant (20). A total of 4,253 protein groups with 25,899 unique peptides and 1,876 protein groups with 17,433 unique peptides were identified using prefractionation and nonfractionated samples, respectively (Fig. S2, Tables S1 and S2). High-pH reversed-phase peptide fractionation allowed a more efficient protein identification than nonfractionated samples. This technique allows to improve protein sequence coverage and low-abundant-peptide identification (21, 22). By implementing stringent filtering criteria, which required a threshold of >70% valid values across the entire dataset, the count of identified protein groups in the fractionated data was reduced to 3,254 and used for further analysis (Table S3).

**Fig. 2.**
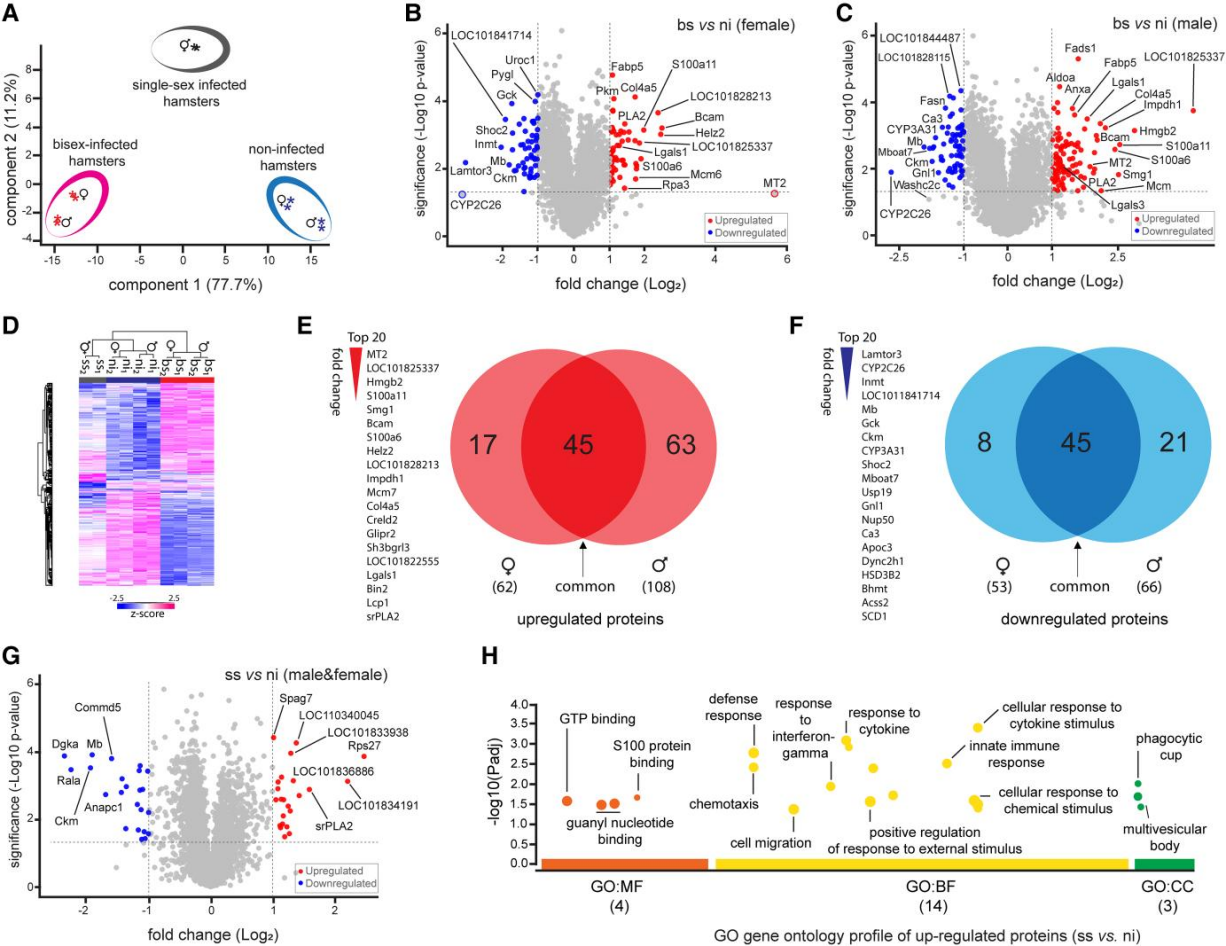
Comparative analysis of hepatic proteomic profiles among analyzed group of hamster livers. The TMT-based dataset with a total number of 3,254 protein groups was used for data analysis. A) PCA across all samples with two technical replicates revealed distinct separation of the liver proteome data between the groups of bs-infected and ni hamsters, suggesting that the infection status (bs) has a significant impact on the liver proteome. B and C) Volcano plots depicting the fold changes (Log_2_) in relation to significance (−Log_10_  *P*-value) of hepatic proteins for bs relative to control (ni) in female and male hamsters (a threshold value of <0.05 is denoted by a horizontal dashed line in each plot). D) Hierarchical clustering analysis of regulated liver proteins between ni and infected (ss and bs) hamsters. E and F) Overlap of significantly different proteins between female and male hamsters, and the top 20 regulated proteins with the order based on fold changes. G) Volcano plot of hepatic proteins; ss relative to controls (ni) of both sexes. H) Manhattan plot of GO profiler query, listing proteins up-regulated between ss and ni samples. The *x*-axis shows the functional terms, grouped as molecular function (MF), biological process (BP), and cellular component (CC).

The high accuracy and sensitivity of the TMT-labeling strategy in quantitative proteomics provided the proper prerequisite for a more precise investigation of differences in the liver proteomes of *S. mansoni*–infected and ni hamsters. To gain a comprehensive understanding of the dataset, with a focus on assessing reproducibility and variability, we employed principal component analysis (PCA) and Pearson's correlation coefficient, as demonstrated in Figs. 2A and S3, respectively. The results highlighted significant disparities in the hepatic proteomes of infected and ni hamsters. This pivotal finding forms a robust basis for further in-depth investigations aimed at unraveling the intricacies of the observed differences.

### Proteomic analyses revealed considerable alterations in the liver proteomes of female and male hamsters infected by *S. mansoni*

To investigate whether different biological conditions (ni, ss, and bs) affect the expression of proteins in livers of *S. mansoni*–infected hamsters, we applied hierarchical clustering based on a multiple-sample ANOVA test. Clustering indicated that the proteome profiles were statistically different between the groups, mainly when focusing on differences between healthy (ni) and disease (bs) clusters (Fig. 2D and Table S3). Furthermore, by comparing the hepatic proteome of the infected (ss and bs) and ni groups, differentially expressed proteins (DEPs) were identified using Student's t test. As a result, 174 (108 up- and 66 down-regulated) and 115 (62 up- and 53 down-regulated) DEPs were identified by comparing liver proteomes of bs vs. ni in male and female groups, respectively (Fig. 2B and C, Tables S4 and S5). In both male and female groups, a subset of the identified altered proteins exhibited a consistent pattern of regulation, with 45 proteins being up-regulated and 45 proteins being down-regulated across both genders (Fig. 2E and F). This shared modulation may underscore potential common molecular responses and highlights proteins that may play pivotal roles irrespective of gender in the context of the investigated conditions. Across all DEPs, representative top-20 with the most remarkable fold changes from each regulated category are also listed in Fig. 2E and F. Remarkably, the number of DEPs was half bigger in males than in females, and it can be proposed that males are more susceptible to *S. mansoni* infection. It is noteworthy that several studies reported immunological dissimilarities between the sexes (23). Females naturally have higher immune responses than males, which may increase the intensity of parasitic infection in males (24, 25). This comprehensive analysis sheds light on the dynamic protein-level shifts occurring in response to *S. mansoni* infection in both male and female hamsters. It is evident that gender significantly impacts the host immune response (26). Further exploration into the precise functions and interactions of these proteins holds the potential to provide invaluable insights into the nuanced dynamics of the host–parasite relationship, elucidating how men and women may exhibit distinct responses to the infection. This, in turn, contributes to a deeper understanding of sex-based differences in susceptibility and response mechanisms.

Furthermore, the up-regulation of some proteins indicated a notable response to the *S. mansoni* infection in both female and male hamsters. Especially, the hepatic levels of metallothionein-2 (MT2), S100 proteins (S100A6 and S100A11), and galectin (LGALS1 and LGALS3) were elevated in hamster livers postinfection with *S. mansoni*. Previous studies have underscored their pivotal roles in mediating liver injuries (27–30). Our observation emphasizes the significance of certain dysregulated proteins in the host’s response to schistosome infection.

### Hepatocellular MT2 is strongly induced by *S. mansoni* infection in both sexes

**Fig. 3.**
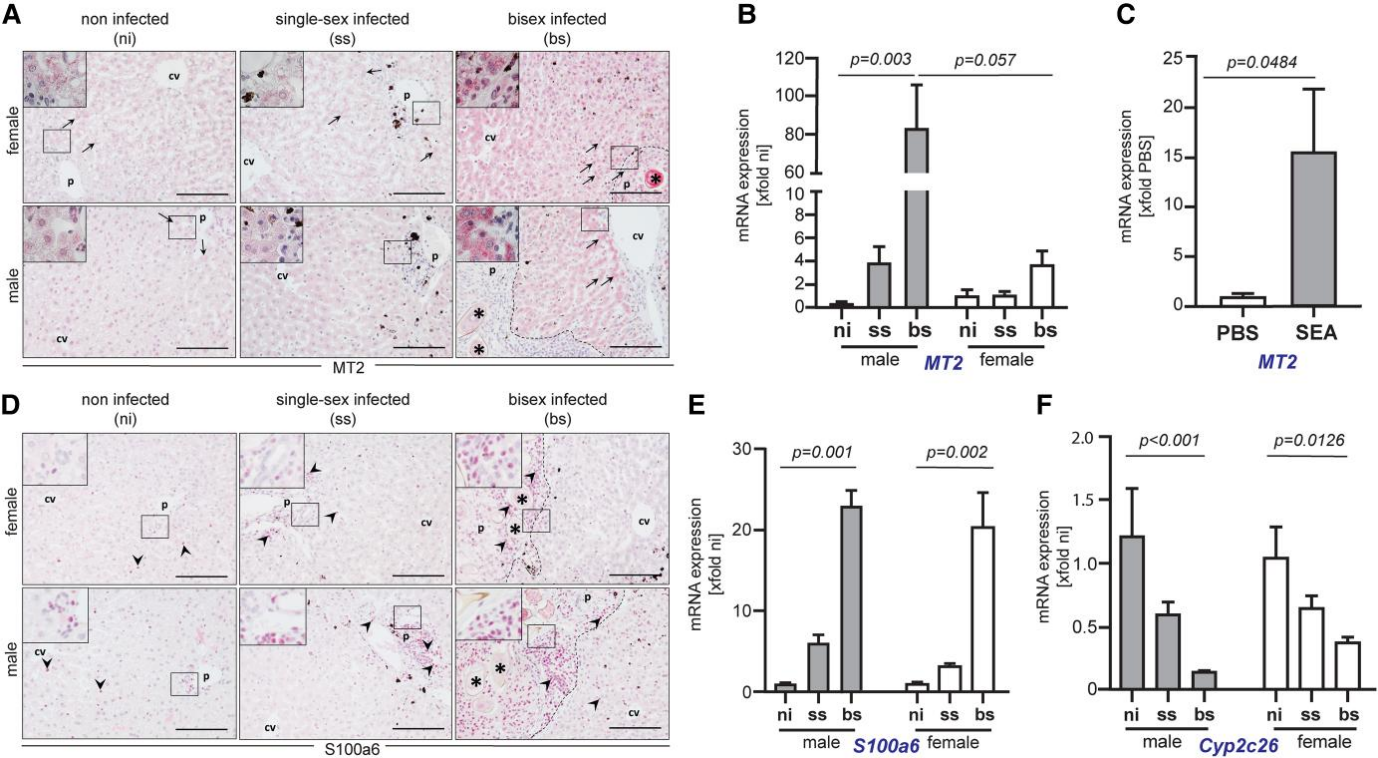
A) IHC staining of hepatic MT2 protein in the liver tissues. B) mRNA expression levels of MT2. C) mRNA expression levels of MT2 in HepG2 cells treated with SEAs or PBS. D) IHC staining of hepatic S100a6 protein in the liver tissues. E and F) mRNA expression levels of S100a6, Cyp2c26 in the livers of infected, and ni control male and female hamsters using the RT-qPCR assay. CV, central vein; PF, portal field; dash line, border of granuloma; arrows, inflammatory infiltration; asterisk, *S. mansoni* egg. Scale bar: 100 µm.

MTs are characterized by their unique structural properties, featuring low molecular weight and a high cysteine content, conferring strong metal binding and redox capabilities. Among the most widely expressed MT isoforms in mammals are MT-1 and MT-2, which are induced in the liver by a diverse array of metals, drugs, and inflammatory mediators (28). Our data revealed up-regulation of MT2 upon *S. mansoni* infection, with a Log_2_-fold change of 5.57 in female livers and 1.86 in male livers, underscoring a substantial increase in expression compared with the control condition (Fig. 2B and C). Notably, in the female sample, we observed an MT2 increase on the boundary of statistical significance (*P* = 0.056), slightly below the conventional threshold of *P* < 0.05 (Fig. 2B). To further validate the significant elevation of MT2 postinfection, we conducted additional functional experiments. Immunohistochemical (IHC) staining of hepatic MT2 also showed marked increase in MT2 expression in liver tissue of bs-infected hamsters compared with ni ones (Figs. 3A and S4A), corroborating the findings from our proteomic analysis. Notably, MT2 protein was detected in the hepatocellular cytoplasm and the nucleus. Moreover, the hepatic expression level of *MT2* mRNA significantly increased in both genders, as confirmed by RT-qPCR analysis (Fig. 3B). These results emphasized the robust response of MT2 to *S. mansoni* infection across male and female hamsters. In contrast to our proteomic results, we observed increased levels of *MT2* mRNA in male hamsters infected with bs, when compared with females (Fig. 3B). This incongruity suggests potential variations in regulatory mechanisms at both mRNA level and posttranslational processes, a well-documented phenomenon (31, 32).

Furthermore, ss infection (without eggs because only paired schistosomes are able to reproduce) showed only a modest increase in *MT2* mRNA levels in both male and female hamsters, which suggests a pivotal role of *S. mansoni* eggs for MT2 overexpression in the liver. To explore the role of deposited eggs in this process, we investigated whether soluble egg antigens (SEAs) of *S. mansoni* were responsible for the increased MT2 expression. Indeed, in vitro experiments with human hepatoma cells revealed that SEA confrontation stimulated *MT2* mRNA expression (Fig. 3C). We have previously demonstrated that SEA induces hepatocellular reprogramming, leading to metabolic exhaustion and a pronounced redox imbalance (12). Due to the distinctive thiolate cluster structure of MT2, it can serve various functions, including participation in zinc homeostasis, protection against heavy metals, defense against oxidative stress, and metabolic regulation through Zn donation (33). Therefore, in addition to the inflammatory triggers, the induction of MT2 in *S. mansoni* infection may occur in response to heightened oxidative stress induced by egg antigen products. Furthermore, the elevated MT2 expression serves as a response to the repair and regeneration of the injured liver (34), a consequence of granuloma formation at the egg sites. As reported, the induction of *MT2* mRNA in zebrafish liver tissue following exposure to silver nanoparticles (AgNPs) also signifies a vital defense mechanism against the potential toxicity of AgNPs (35). This adaptive response is closely linked to the observed pathological effects, including oxidative stress, DNA damage, and apoptosis. Overall, the induction of MT2 is a crucial component of cellular defense strategy, working in tandem with other metabolic pathways to safeguard liver against the adverse effects, such as schistosoma eggs. However, our data showed that the MT2 protein level was over 2-fold changed (Log_2_) in females than in males (Fig. 2B and C). This conspicuous gender discrepancy likely stems from the heightened concentration of iron in female liver tissue (36) or potentially influenced by sex hormones (37). Iron is widely acknowledged for its pivotal role in catalyzing the production of reactive oxygen species and triggering oxidative stress (36), which may synergistically intensify the impact of oxidative stress induced by schistosome infection.

### *S. mansoni* infection induced up-regulation of proteins with immunomodulatory capacity in host liver

Among others, the proteomic analysis revealed a notable up-regulation of a member of the S100 protein family, here specifically S100a6 and 11, in the livers of bs-infected hamsters and without host gender disparity (Fig. 2B and C). S100 proteins are calcium-binding proteins and, as such, are involved in a multitude of metabolic pathways, playing pivotal roles in cellular processes, such as proliferation, apoptosis, differentiation, and inflammation (38). IHC staining depicted an increased presence of nonparenchymal cells with S100a6-positive nuclei in the livers of bs-infected hamsters (Figs. 3D and S4B). S100a6 protein levels were predominantly elevated within the granuloma and around the portal tract. The hepatic mRNA expression assay further demonstrated a substantial increase in *S100a6* mRNA expression levels, regardless of gender, in bs-infected hamsters (Fig. 3E), corroborating the findings from the proteomic data. As shown in Fig. 3E, hamsters infected with ss cercariae exhibited a slight elevation in *S100a6* mRNA level. This observation corresponds to our finding with MT2 and again suggests a decisive role of *S. mansoni* eggs in the liver, which responds to the infection by up-regulating the expression of both proteins. S100a8 and S100a9 proteins have also been identified in areas of neutrophil accumulation in liver tissue infected by *S. japonicum* (39), and they are implicated in neutrophil recruitment and the production of pro-inflammatory cytokines (40–42).

The observed up-regulation of galectin proteins in livers of bs-infected hamsters represents another piece of the infection and pathology puzzle. Galectins constitute a class of proteins with high affinity for β-galactoside-containing oligosaccharides. They have garnered attention owing to their diverse immunoregulatory functions (43). High levels of galectin-1 and galectin-3 expression have been documented in the context of *Schistosoma* infections (10, 44). Our proteomic analysis confirmed the up-regulation of galectin-1 protein expression in both male and female hamsters (Fig. 2B and C). However, galectin-3 induction was observed exclusively in the livers of infected male hamsters, emphasizing potential sex-related dissimilarities following *S. mansoni* infection. Thus far, both of these proteins have demonstrated multifaceted functions within infected host organs. For instance, galectin-1 exhibits anti-inflammatory properties, influences apoptosis in T helper cells, and promotes tissue fibrosis (45, 46). Conversely, galectin-3 plays a pro-inflammatory role by binding to the antigenic glycan on the *Schistosoma* egg surface, facilitating antigen recognition by macrophages, and modulating immune and inflammatory responses (43–46). Despite their seemingly conflicting functions, they may work in tandem to regulate inflammation and maintain immune homeostasis (43).

### *S. mansoni* infection induces inhibition of critical signaling pathway components in host liver

Proteins such as cytochrome P450 (CYP), creatine kinase (CKM), phosphotransferase (Gck), apolipoprotein (Apoc2,3), and glutathione-*S*-transferases (GSTs) were found to be down-regulated in the livers of bs-infected hamsters. These factors are involved in critical metabolic processes, including xenobiotic biotransformation (CYPs and GSTs), lipid modulation (Apoc), cellular energy metabolism (CKM), and glycolytic processes (Gck) (47–50). The proteomic analysis detected the Cyp2c26 isoenzyme as the most strongly down-regulated target in *S. mansoni* infection for both sexes, exhibiting a fold change of −2.5 in males and −3.1 in females. However, it is noteworthy that in females, the down-regulation of Cyp2c26 narrowly misses the conventional *P*-value threshold (<0.05), displaying a *P*-value of 0.059 (Fig. 2B and C). Therefore, we performed RT-qPCR, analyzing the Cyp2c26 mRNA expression levels of ni-, ss-, and bs-infected livers. The results revealed hepatic suppression of Cyp2c26 mRNA expression by *S. mansoni* infection confirming the proteomic analysis (Fig. 3F). Depleting CYPs and GSTs promotes oxidative stress and changes the capacity of the liver to detoxify many endogenous and exogenous compounds (51), which may be directly associated with *S. mansoni*–induced pathology (52). It has been reported that CYP is down-regulated during chronic murine schistosomiasis, which may be associated with the induction of Th2 inflammatory cytokines (53). The regulation of these enzymes can change the therapeutic potential of drugs administered to infected patients, which they metabolize to around 80% of prescribed drug (54, 55).

### Single-sex infection with *S. mansoni* cercariae drives moderate immune responses

The proteome comparison of ss-infected hamsters vs. ni controls showed relatively few DEPs (Fig. 2G and Table S6). It has been defined that the livers of ss-infected animals lack mature eggs, which results in the absence of inflammation or granulomatous lesions (56). However, in rare cases, nonfertilized egg-like structures have been observed in ss-infected hamsters, which can be accompanied by host reactions (57). Earlier studies also documented that ss infections led to an increase of liver weight, slowed hypersensitivity, increased the number of peripheral blood cells and antibody responses, and induced specific immune responses, leading to pathological changes in the liver (58–61). In our experiment, inflammatory infiltrates indicated moderate inflammation in the livers of ss hamsters (Figs. 1A and S1) and slight up-regulation of a few proteins linked to immune responses (Fig. 2H). Furthermore, we observed several moderately down-regulated proteins in livers of ss-infected hamsters (Fig. 2G), such as COMM domain-containing protein 5 (COMMD5) and diacylglycerol kinase (DgKa). COMMD5 is involved in immune responses and inflammation (62). DgKa is part of pathways regulating T-cell receptor signaling to maintain normal immune system homeostasis (63). Recently, a model involving ss cercariae for human infection has been developed as a vaccination strategy for schistosomiasis (64). In this study, some infected volunteers exhibited acute schistosomiasis syndrome, leading to elevated liver enzymes. Thus, it is important to investigate in greater detail how liver functions and metabolites may undergo slight alterations in response to ss infections.

### *S. mansoni* infection modulates liver metabolic pathways

**Fig. 4.**
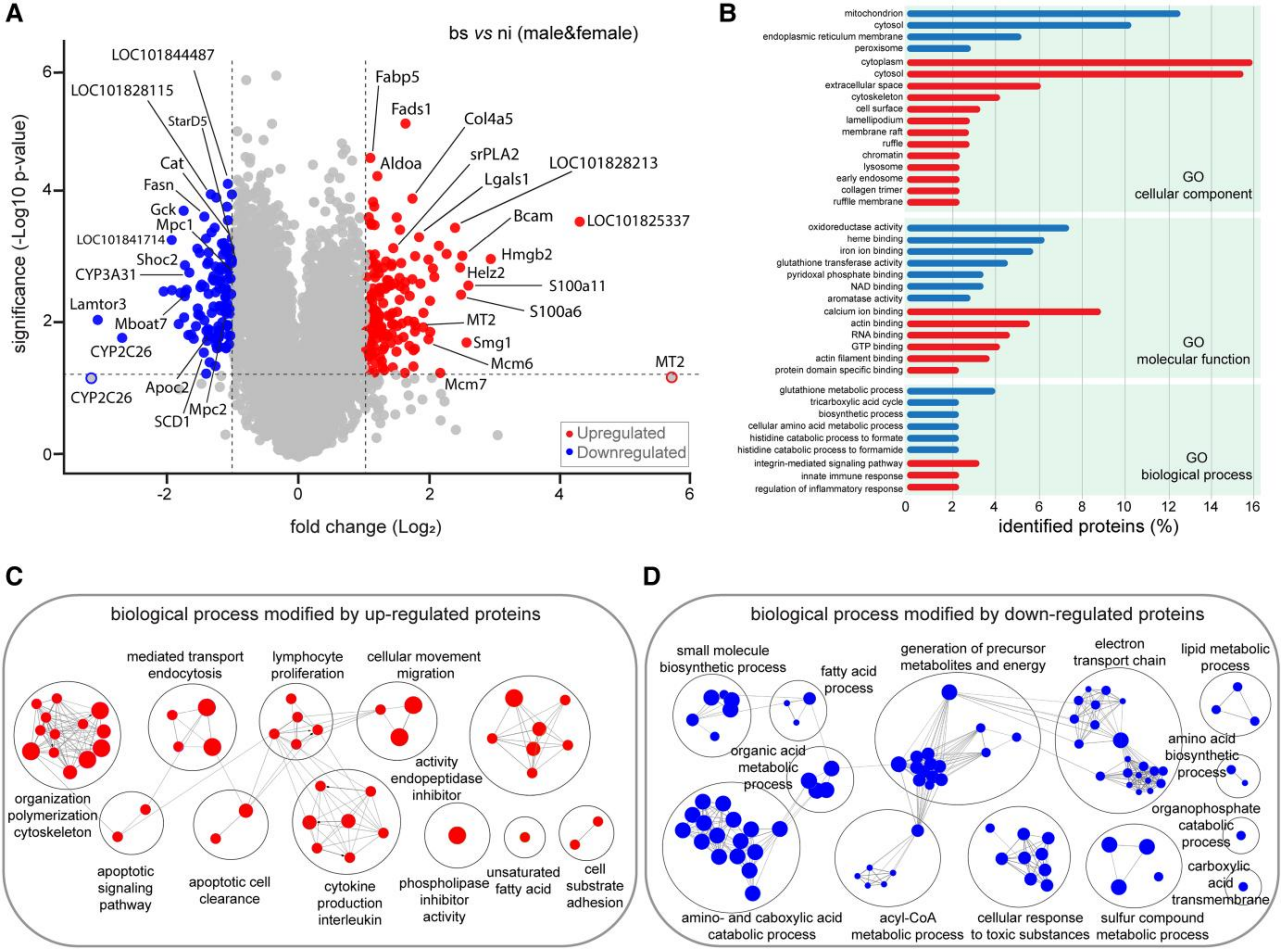
A) Volcano plots depicting the fold change (Log_2_) relative to significance (−Log_10_  *P*-value) of hepatic proteins for bs relative to control (ni) by combing data related to female and male hamsters; a horizontal dashed line represents the significance threshold, set at *P* < 0.05, indicating the cutoff for statistical significance. B) GO functional analysis of DEPs, using DAVID to extract biological meaning from dysregulated proteins. C and D) ClueGO and Cytoscape GO analyses show pathways associated with dysregulated proteins. The edges offer the connectivity between each node, and the size of the nodes depends on the number of grouped proteins.

Schistosomiasis goes on with alterations in hepatic protein expression when parasitic eggs are deposited in the liver (9, 10, 65, 66). In our proteomic study, through the integration of data from both genders, we observed that over 200 proteins exhibited statistically significant regulation when comparing samples from bs-infected hamsters with those without infection (Fig. 4A, Tables S4 and S5). This exhibits a fundamental remodeling of the hepatic proteome of *S. mansoni*–infected hamsters. Most of the dysregulated proteins localized in the cytosol, cytoplasm, mitochondria, and endoplasmic reticulum membrane, where they primarily rearrange various critical metabolic pathways, such as cellular immune response, glutathione metabolic processes, and amino/carboxylic acid metabolic processes in response to the infection (Fig. 4B).

To define the biological functions in which DEPs are involved, we performed a gene ontology (GO) pathway enrichment analysis of the regulated proteins (Fig. 4C and D), using the ClueGO plugin (67) from Cytoscape (68). The results showed that the up-regulated proteins are particularly associated with immune responses. They were enriched in pathways related to lymphocyte proliferation, cytokine production, cytoskeleton reorganization, apoptotic signaling, and cell clearance. These findings are consistent with the observation that *S. mansoni* eggs induce a variety of immunological responses in the liver (e.g. increasing lymphocyte and cytokine production) (69) which may ultimately drive extensive tissue fibrosis (elevation of cytoskeleton/actin organization pathways) in the way of granuloma formation. A genomic study of mouse livers with granulomas and fibrosis caused by *S. japonicum* eggs is also in harmony with these observations (39). Besides, schistosome eggs from *S. mansoni* and *S. japonicum* can inhibit hepatic stellate cell activation and induce immunocyte apoptosis (70–72), which may explain the increased apoptotic and cell clearance pathways in livers of *S. mansoni*–infected hamsters. Mainly, modifying immune cell responses can regulate host metabolic homeostasis as part of health and disease (73) and alter the metabolism of the affected organ of the host, particularly disrupting amino acid, lipid, and energy metabolisms (74–80), which is the case for *S. mansoni* infection.

Ontology assortment, on the other hand, showed that the down-regulated proteins were linked to many metabolic pathways. They enriched in biological processes such as energy derivation by oxidation of organic compounds, generation of precursor metabolites and energy, electron transport chain, amino and carboxylic acid catabolic process, fatty acid process, organic acid metabolic process, small molecule biosynthetic process, and cellular response to toxic substances (Fig. 4D). Some of these findings agree with those reported for *S. mansoni* and *S. japonicum* infections, and they most likely point to a decline in liver function and accompanying increase of tissue damage (9, 74, 81).

### *S. mansoni* infection affected hepatic lipid compositions and energy production

Our recent study showed that *S. mansoni* eggs induced tissue damage in the host, disrupting lipid and carbohydrate metabolism, ultimately leading to oxidative stress within the host’s parenchyma (12, 13, 82). Additionally, here we found that the infection caused by *S. mansoni* eggs can disturb pathways associated with carbohydrate, fatty acid, and lipid metabolism/synthesis in the liver (Fig. 4D, Tables S7 and S8). Our proteomic data exhibited a dysregulation in the expression level of hepatic enzymes (e.g. *Fabp5*, *Fads1*, srPLA2, *Fasn*, *Apoc2*, *Cat*, *LOC101841714*, and *Mboat7*) involved in lipid metabolism in livers of bs-infected hamsters when compared with the ni control (Fig. 4A and Table S8). Similar findings have also been reported in independent studies (49, 79, 80). To discover the metabolic consequences of these protein dysregulation in more detail, we also conducted MALDI-MSI and LC-MS/MS lipidomics of liver samples belonging to the bs and ni groups (Fig. 1). Untargeted lipidomics revealed significant alterations in the hepatic lipid content of both female and male infected hamsters (Fig. S5 and Table S9). A total of 1,209 lipid species belonging to 24 lipid classes were quantified by combining the results of positive- and negative-ion mode (Table S9). The infection significantly altered the hepatic lipidome, affecting nearly all the main lipid classes (Fig. S5). Indeed, given the observed down-regulation of enzymes involved in fatty acid biosynthesis (such as *Apoc2*, *Fasn*, and *SCD1*) in the proteomic data (Fig. 4A), we anticipated an imbalance in the hepatic lipid contents of livers of bs-infected hamsters.

**Fig. 5.**
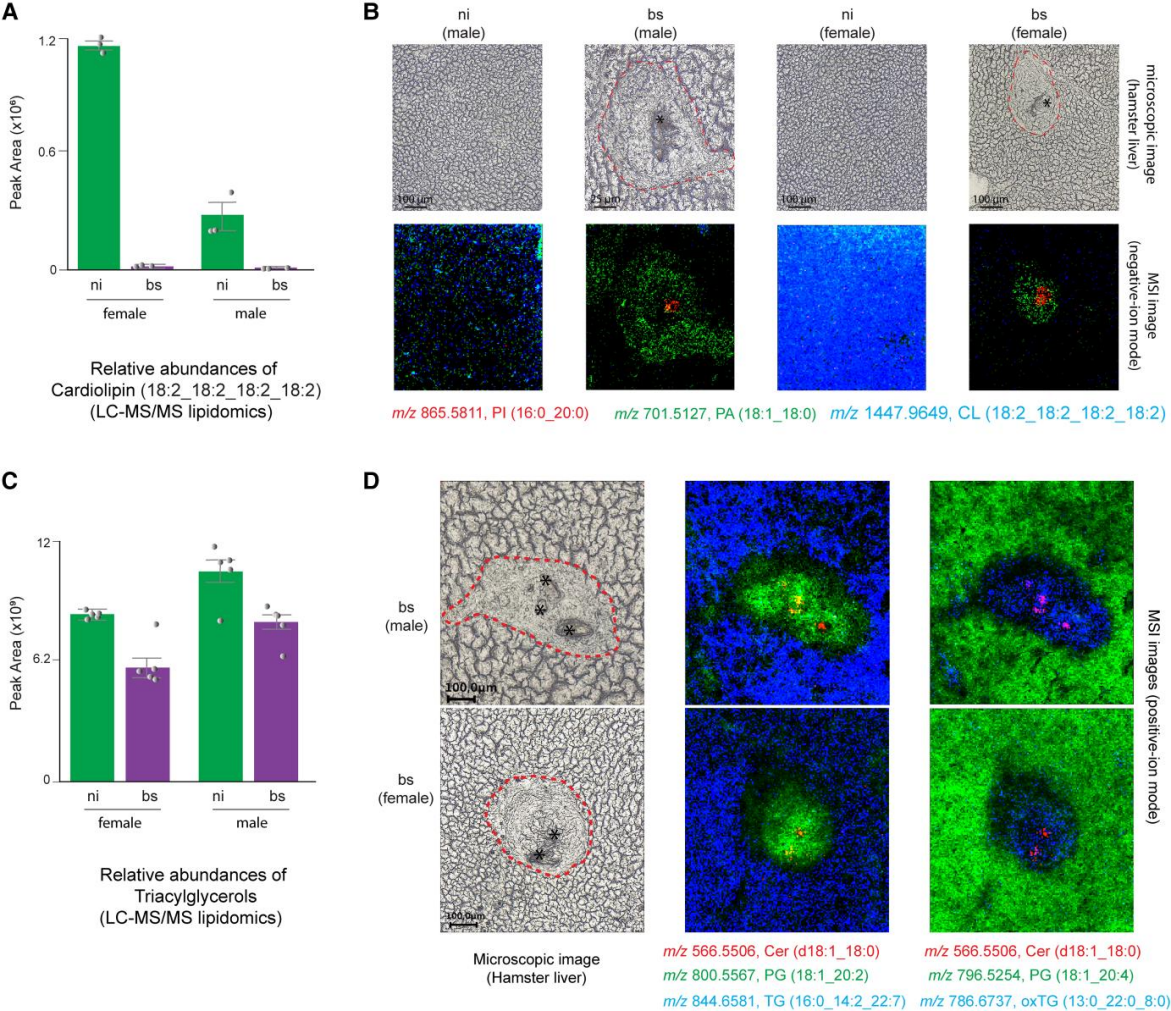
Representative comparative hepatic lipid analysis of *S. mansoni*–infected hamsters. A) The level of hepatic CL species decreased significantly in livers of bs-infected hamsters. B) MALDI-MSI analyses visualized the distributions of targeted lipid species in liver sections of the ni control compared with bs infection. C) The LC-MS/MS quantification of TG lipid species revealed depleted hepatic levels of TG in bs-infected hamsters. D) MALDI-MSI analyses showed different distributions of targeted TG species in the livers of hamsters infected with bs compared with ni controls. CL, cardiolipin; PA, phosphatidic acid; PI, phosphatidylinositol; Cer, ceramide; PG, phosphatidylglycerol; TG, triacylglycerol; Red dashed line, border of granuloma; asterisk, *S. mansoni* egg.

We further investigated whether this reorganization of the hepatic lipidome in infected hamsters influenced specific membrane lipids. A lipid-reaction analysis method (83) was used to estimate lipid pathway activity, relying on the consistent levels of lipids as determined by lipidomic data. In particular, there was a considerable suppression in the activity of reactions and pathways involved in synthesizing cardiolipin (CL; Figs. 5A and S6). CLs, categorized as phospholipids, are exclusively localized within mitochondrial membranes. They have diverse functions, such as the regulation and organization of the electron transport chain (84). As illustrated in Fig. 5B, the application of MALDI-MSI allowed for the visualization of the specific CL species (18:2_18:2_18:2_18_2) within liver tissue sections from both bs-infected and ni groups. This observation substantially complements the insights garnered from LC-MS/MS data, underscoring the critical involvement of CLs in the hepatic response to the infectious challenge. CL anomalies in the mitochondrial inner membrane are a potential cause of mitochondrial dysfunction induced by chronic inflammation and concurrent oxidative stress (85). Furthermore, the down-regulation of key enzymes associated with the biological function of mitochondrial transmembrane transport, specifically *Mpc1*, *Mpc2*, and *Ccdc51*, strengthens the hypothesis of mitochondrial dysfunction during the infection (Fig. 4A and Table S8). Thus, a decrease in CL levels may subsequently impair mitochondrial energy production, influencing cellular processes and tissue health (86). Hence, maintaining adequate CL levels is crucial for cellular and organ health, particularly in tissues with high energy demands like the liver. As indicated by lipid pathway analysis, the conventional route for CL biosynthesis involves the precursor molecule phosphatidylglycerol (PG) (87). Intriguingly, pathways degrading or converting phosphatidic acid (PA) to PG were significantly active, and the lipidomic data additionally disclosed an elevated overall abundance of PG in livers of infected hamsters (Fig. S5). The concurrent decrease in CL and increase in PG observed during the infection may be attributed to a range of intricate regulatory mechanisms. While it would seem logical for an increase in PG to lead to an increase in CL production during the CL synthesis reaction, a recent study has elucidated that an excess of PG triggered CL hydrolysis, resulting in decreased CL concentration, and PG supplementation was found to help suppress inflammation (85). However, in-depth empirical investigation of the precise reasons driving these phenomena in *S. mansoni* infection is required and subject of future studies.

Consistent with the shift toward negative synthesizing CL, there was a significant reduction observed in the quantities of neutral lipid triacylglycerols (TGs; Fig. 5C), aligning with the findings of our prior investigation (12, 82). Down-regulation of enzymes involved in the biological function of TG metabolic processes (e.g. *cat*, *Apoc2*, and *Mboat7*) was in line with the results (Fig. 4A and Table S8). The decrease in TG levels could be attributed to a reallocation of energy resources. Infection often triggers a shift in energy usage toward immune responses and away from processes, like lipid storage (88). Furthermore, our studies have demonstrated that *S. mansoni* eggs possess the ability to take up lipids, exhibiting a specific preference for neutral lipids, from hepatocytes (12). In this context, our observations revealed a heterogeneous distribution of TG species in the livers of bs-infected hamsters. The findings from MALDI-MSI analysis indicated distinct localization patterns for TG species. They were predominantly found either within the granuloma, particularly within the eggs, or in the extracellular region surrounding the granuloma (Fig. 5D). Notably, this phenomenon also extends to lysophospholipids with a substantial portion exhibiting a tendency to concentrate within the eggs (Fig. S7). The lipidomic result revealed enhanced hepatic levels of most of them (e.g. LPC, LPS, LPG, and LPE) in bs-infected animals (Fig. S5). These molecules are distinct from their parent lipids, originating from membrane lipid hydrolysis, and they play crucial roles in both structural and signaling functions, contributing significantly to cellular and organismal physiology (89). Despite their importance, our understanding of TGs remains limited. Unraveling their biosynthesis, transport, and functions holds promise for clinical therapeutics (89). Further research is needed to uncover their physiological significance, which could illuminate novel signaling pathways and broaden our understanding of host–parasite interaction at the liver-stage level.

Another notable observation was the diminished expression of the endoplasmic reticulum (ER) stress protein, Steroidogenic Acute Regulatory Domain 5 (StarD5), in livers of bs-infected hamsters compared with the control group (Fig. 4A). StarD5 plays a crucial role in maintaining hepatic cholesterol homeostasis, and its perturbation has been shown to contribute to apoptotic and hepatocellular carcinogenesis (90, 91). This alteration implies an augmented activation of the ER-stress response, leading to subsequent perturbations in liver cholesterol and cholesterol ester content, along with a simultaneous increase in intracellular neutral lipids, such as di- and mono-acylglycerols, as observed here (Fig. S5) (12). Nevertheless, dysregulation in cholesterol homeostasis is a hallmark in the pathogenesis of numerous diseases, suggesting a potential association between *S. mansoni* infection and various hepatic disorders, including liver cancer. It is important to note, however, that the available data regarding the precise role of *S. mansoni* in the process of carcinogenesis are conflicting and often lack conclusive evidence of causality. This underscores the need for further comprehensive research to elucidate the intricate relationship between this parasitic infection and the development of hepatic disorders (92).

In general, the liver is a key player in the physiological control of the body's energy homeostasis, mainly by regulating glucose and lipid metabolism. Our results showed that *S. mansoni* infection caused cellular energy stress, altering the expression of glucose and lipid metabolism enzymes in bs livers. The infection impaired energy production by affecting energy metabolic processes including, e.g. fatty acid β-oxidation, tricarboxylic acid cycle, mitochondrial electron transport and ATP synthesis (Fig. 4D and Table S8). The down-regulation of liver energy homeostasis has been linked to a steady loss in health and an increased probability of developing more serious disease symptoms including liver failure or tumor initiation and metabolic illnesses. Deeper insight into schistosomiasis-induced aberrant metabolic activities of the liver requires additional clinical and experimental studies (in vivo and ex vivo) in the future. Among others, they may lead to novel biomarkers for the early diagnosis of hepatic abnormalities and predictors of the progression of schistosomiasis-associated liver failure.

## Conclusion

In this study, we employed advanced MS-based proteomics and lipidomics to meticulously analyze the hepatic protein and lipid profiles within *S. mansoni*–infected and ni hamster models. Our investigation yielded unprecedented insights into the composition of these biomolecules, a critical foundation for comprehending the clinical and pathological implications of schistosomiasis. The results show evident changes in protein and lipid compositions in the liver of hamsters during *S. mansoni* egg accumulation, indicating metabolic disruption and tissue damage. Typically, up-regulated proteins could be associated with immune responses, while down-regulated ones involved metabolic pathways, providing a new avenue for future studies. Notably, gender-specific responses were observed, with males showing higher susceptibility, supported by a greater number of DEPs compared with females. Key proteins, such as MT2, S100 family, and galectins, were found to be significantly regulated. These proteins may play vital roles in mediating parasite-induced liver injuries. We have detected significant changes in the expression levels of key enzymes involved in critical signaling pathways, such as CYP, CKM, and GST. These observed changes have the potential to exert a significant influence on drug metabolism. Nevertheless, it is essential to emphasize that our study primarily focused on the profiling of liver proteome and lipidome, and this particular aspect will be extensively considered in our future research. Moreover, extensive reorganization of hepatic metabolic pathways and lipid perturbations was discovered, emphasizing the critical role of lipids in host–parasite interaction at the egg-liver stage and their potential influence on cellular processes and tissue health. In summary, our study sheds new light on the intricate interplay between host liver and *S. mansoni* eggs and, as such, may be stimulating for further research into the mechanisms underlying schistosome-induced hepatopathy and new therapeutic strategies.

## Materials and methods

### Chemicals

All the reagents used in the study were at least of analytical grade and purchased from Sigma-Aldrich or mentioned in the respective protocols.

### Ethics statement

Animal experiments described in this study were carried out following the European Convention for the Protection of Vertebrate Animals used for experimental and other scientific purposes (ETS No 123; revised Appendix A). Furthermore, all experimental protocols were approved by the Regional Council (Regierungspraesidium) Giessen (V54-19 c 20/15 h 02 GI 18/10 Nr. A 14/2017).

### Cell culture experiments

HepG2 cells (stock ordered in 2019, CLS # 330198, expanded and stored as cryostocks for consistent quality in culture for up to 10 passages per cryostock) were stimulated with 15 µg/mL SEA for 6 h.

### Quantitative RT-PCR

mRNA isolation, transcription, RT-qPCR, and data analysis were performed as described previously (93).

### Immunohistochemistry

IHC detections were performed as described previously (94).

### Statistical analysis

The present study is of an exploratory nature. The study was performed with an existing number of cryo-preserved organs that were not required for the maintenance of the parasite life cycle. Statistical analysis was performed with Kruskal–Wallis test and Student's t test using GraphPad Prism version 5.3.1 (GraphPad Software, LLC, d.b.a Dotmatics). Because of the exploratory nature of the study, no further adjustment for *P*-values was performed.

### Hamster infection and sample groups

Syrian hamsters (*M. auratus*) were used as final hosts for *S. mansoni* life cycle, and snails (*Biomphalaria glabrata*) were used as intermediate hosts. Both snails and hamsters were bred in-house (Biomedical Research Center Seltersberg, Giessen, Germany), and a Liberian strain of *S. mansoni* was used for infection (95). For this study, female and male hamsters were separated into three main groups, including ni hamsters as controls (ni; *n* = 12), ss-infected hamsters of *S. mansoni* cercariae (*n* = 12), and bs-infected hamsters of cercariae (*n* = 12). We employed 1,750 cercariae for bs infections and 2,500 for ss infections. The infection method involved the bathing/paddling technique, consisting of a 30-min preincubation in 30°C water (0.5 cm high in a plastic container) followed by an additional 30 min in 30°C water with cercariae exposure (96). Prior to infection, clonal cercariae (ss) underwent sex determination through “sexing PCR” (97). Equal numbers of hamsters were infected with ss females and males, with subsequent regular counting of worm populations. In bs infections, worm recoveries averaged 80–160 couples per hamster, while ss infections yielded 100–200 individual males or females. The bs infections were carried out at the age of 8 weeks and were maintained for 46 days, and ss infections 67 days to ensure a complete maturation of the worms; females need longer to grow and develop in the absence of male partners. Liver tissue of hamsters was harvested upon worm perfusion and rinsed with phosphate buffered saline (PBS) to remove residual blood. The liver tissue was shock-frosted in liquid nitrogen, and 70% of the whole liver was grounded under liquid nitrogen. The samples were grouped and immediately transferred to a −80°C freezer for further analysis. Liver sections were stained with H&E to visualize hepatica architecture and inflammatory infiltration.

### Protein sample preparation

Samples were placed into individual Protein LoBind tubes (Eppendorf, Hamburg, Germany), containing T-PER tissue protein extraction reagent (pH 8.0) accompanied by protease inhibitor (Thermo Fisher Scientific, Rockford, IL, USA) and homogenized for complete cell lysis. Supernatants were collected, and the protein concentration was determined using a standard Bradford assay (Bio-Rad, Hercules, CA), with bovine serum albumin used as reference. An amount of 100 µg protein per group was transferred into a new tube and adjusted to a final volume of 100 µL with 100 mM triethyl ammonium bicarbonate (TEAB). The samples were first reduced with 200 mM tris(2-carboxyethyl)phosphine at 55°C for 1 h and then alkylated with 375 mM iodoacetamide for 30 min in a dark place at room temperature. The proteins were precipitated by methanol/chloroform extraction protocol and dried in a vacuum. The lyophilized protein pellets were resuspended with 100 µL of 50 mM TEAB and digested with Trypsin/Lys-C mixture (Mass spec grade, Promega, Madison, WI, USA) at 25:1 protein:protease ratio (w/w) and incubated overnight at 37°C. The digestion reaction was stopped after 16 h, adding trifluoracetic acid (TFA) to 0.5%, and samples were dried and stored at −80°C freezer.

### TMT labeling and high-pH reverse-phase peptide fractionation

Peptides were resuspended in 100 mM TEAB, and their concentration was determined using a NanoPhotometer (Implen, Munich, Germany). TMT 6-plex reagents were used for labeling 100 µg protein digest, according to the manufacturer's protocol (TMT Mass Tagging Kit; Thermo Fisher Scientific, Rockford, IL, USA). Briefly, 41 µL of TMT label reagents were added to each 100 µL sample and incubated for 1 h at room temperature. Next, 8 µL of 5% hydroxylamine was added to the samples and set for 15 min to quench the reaction. Finally, samples were combined at equimolar amounts in a new tube and subjected to fractionation before nLC–MS/MS analysis. To decomplex the peptide mixture and increase peptide identification, the TMT-labeled sample was fractionated using the High-pH Reverse-Phase Peptide Fractionation Kit (Pierce, Thermo Fisher Scientific, Rockford, IL, USA), following the protocol provided by the manufacturer.

### nLC-MS/MS measurements

The TMT-labeled samples were reconstituted in 5% formic acid and subjected to separation using an UltiMate 3000 RSLCnano system (Thermo Fisher Scientific, Bremen, Germany) coupled with a Q Exactive HF-X Orbitrap mass spectrometer (Thermo Fisher Scientific, Bremen, Germany). Each sample, comprising 1 µg of peptides, underwent loading on an Acclaim PepMap trap column (100 µm × 2 cm, C18, 5 µm, 100 Å) and subsequent separation on an Acclaim PepMap RSLC analytical column (75 µm × 150 mm, C18, 2 µm, 100 Å). A 160-min mobile phase gradient, ranging from 5 to 45% acetonitrile/0.1% formic acid, was employed for peptide separation, with a flow rate set to 300 nL/min. The mass spectrometer operated in data-dependent acquisition (top-15 DDA) mode with specified parameters for full MS scans, including a mass range of *m/z* 350–1,400, mass resolution of 120,000 (@ *m/z* 200), automatic gain control (AGC) target of 3 × 10^6^, maximum injection time (IT) of 50 ms. For MS/MS scans, the mass resolution was set at 45,000 (@ *m/z* 200), AGC target of 1 × 10^5^, maximum IT of 96 ms, isolation window *m/z* ± 1, dynamic exclusion for 30 s, and normalized collision energy (NCE) of 32.

### Proteomics data processing

Raw MS files were processed using MaxQuant software, version 2.1 (20). All files were searched with the Andromeda search engine against the *M. auratus* Uniprot database (downloaded on January 2022) and common contaminants (provided with MaxQuant) with forward and reverse sequences. The reporter ion masses and mass tolerance were updated on the software, and the reporter ion of 131 was assigned as a reference channel for normalization. Next, reporter ion MS2 was selected, and normalization was given to the weighted ratio to the reference channel and isobaric matching between the runs (98). The search was configured with fixed modification of carbamidomethyl cysteine and dynamic modifications of methionine oxidation and N-terminal acetylation. The FDR was set to <0.01 for peptide and protein identification. Search results generated by MaxQuant were analyzed in Perseus software (20). Protein groups known as contaminants and reverse were initially removed and data logarithmized for the next steps. The protein groups were filtered with a missing value of >70% in total across all channels. Next, missing values were imputed by employing a sampling approach from a normal distribution. The width parameter was set to 0.3, while the down-shift parameter was set to 1.8, aligning with the default values in Perseus. PCA was performed on the imputed value after the median subtraction of the proteome data.

### Hierarchical clustering, profile comparison, and network analysis

For data processing workflow, we conducted hierarchical cluster analysis and compared liver protein profiles for all samples using Perseus software. For in-depth analysis of the refined dataset, multiple-sample tests were performed, specifically employing an ANOVA test. To enhance the reliability of our results, we applied a truncation-based Benjamini–Hochberg FDR control set at 0.05. Subsequently, significant outcomes from the ANOVA analysis were elucidated through the generation of a heatmap. This process involved reprocessing the data, utilizing the Fisher exact test, and scaling the data based on *Z*-scores to facilitate meaningful visual comparisons. VolcaNoseR was used to generate the volcano plots based on the processed data, setting Log_2_ of the fold change on the *x*-axis and minus Log_10_ of the *P*-value on the *y*-axis (99). For network analysis and pathway generation, the ClueGO plugin in Cytoscape software version 3.9.1 was used to establish the biological interpretation of the regulated proteins (67, 68). In addition, the online web tools gProfiler and DAVID were used to perform functional enrichment analysis on an input protein list (100, 101).

### MS-based lipidomic and data analysis

Around 100 mg of liver tissue from each biological sample (male and female bs and ni) were homogenized using a laboratory nano ball mill (Fritsch pulverisette 23, Germany). Before lipid extraction, SPLASH LipidoMIX (Avanti Polar Lipids, Inc.) deuterium-labeled internal standards were spiked in samples to estimate the concentration of annotated lipid species. Lipid extraction was conducted using a cold mixture of methanol and methyl-tert-butyl ether (MTBE) in a ratio of 1:3 (v/v). To outline the procedure, 330 µL of cold methanol was introduced to each sample, followed by 5 min of vortexing and a 10-min incubation on ice. Subsequently, 1 mL of frigid MTBE was incorporated into the solution, followed by vortexing and a 5-min sonication period with the use of ice. The solution underwent a 1-h incubation on the Thermomixer (Eppendorf Thermomixer C) at 2°C with a rotation speed of 950 rpm. Following this, 250 µL of cold water was introduced, incubated for 5 min, and subjected to cold centrifugation (Beckman Coulter, Krefeld, Germany) for 10 min at 12,000 rpm. The resultant upper organic layer, presumed to contain the lipids, was carefully transferred into a new precooled glass vial. Subsequently, the organic layers were evaporated using a nitrogen flow, and the resulting dry lipids were stored at −80 °C until further MS-based lipidomic analysis. Additionally, a pooled liver QC sample was generated by combining equal volumes from each biological study sample.

The lipid samples were analyzed in five technical replicates using the following procedure: The samples underwent separation on an analytical column, Kinetex C18 (Phenomenex, Torrance, CA, USA), with dimensions of 2.1 × 100 mm, a particle size of 2.6 µm, and a pore size of 100 Å. This column was connected to a Thermo Scientific Dionex UltiMate 3000 UHPLC system. The mobile phase A consisted of a blend of ACN/H_2_O (60:40), while mobile phase B was composed of IPA/ACN/H_2_O (90:8:2). Both mobile phases included 10 mM ammonium formate and 0.1% formic acid. The flow rate was maintained at 250 µL/min, and the gradient elution initiated at 20% mobile phase B. It increased to 30% B over 4 min, 45% B over the next 2 min, 60% B over 4 min, 65% B over the subsequent 4 min, and was maintained for an additional 4 min. A steep increase to 90% B occurred over 13 min, followed by column re-equilibration with 20% B for 10 min before the next injection. Ionization of the samples in both positive- and negative-ion modes was performed using a heated electrospray ionization source (HESI II) connected to the Q Exactive HF-X Orbitrap mass spectrometer. The ion source settings were fine-tuned as follows: a spray voltage of ±3 kV, a source temperature of 325°C, a capillary temperature of 300 °C, and a sheath gas flow of 35 (30 for negative polarity). Each sample underwent measurement in both ion polarities through top-10 DDA with specific parameter values during full MS scan, including a mass range of *m/z* 350–1,200, a mass resolution of 60,000 (@ *m/z* 200), AGC target set at 5 × 10^6^, maximum ion IT of 75 ms, and MS/MS scans with a mass resolution of 60,000 (@ *m/z* 200), AGC target of 5 × 10^6^, maximum IT of 175 ms, isolation window of *m/z* ± 1, dynamic exclusion for 6 s, and stepped NCE levels of 20–25–30.

For lipid data analysis, LipidSearch 5.1 software (Thermo Fisher Scientific) was employed for both lipid identification and relative quantitation. The parameters utilized for the selected lipid classes, as well as the data processing and filtering steps, are outlined in Fig. S8. Within each biological group, consisting of five replicates for positive-ion mode and three for negative-ion mode, potential lipid species were identified independently from both positive- and negative-ion adducts. The resultant data were then integrated within a defined chromatographic time window. This integration process involved consolidating positive- and negative-ion annotations into a unified lipid annotation results table. To illustrate, the quality and reproducibility of base peak chromatograms derived from a pooled hamster liver extract with five technical replicates were assessed, as depicted in Fig. S9. The concentrations of lipid species were estimated by considering the internal standard concentration and calculating the peak area ratio of the analyte to the internal standard. Furthermore, for statistical analysis and data visualization of the lipidomic dataset, R version 4.2.1 was utilized, employing the ggplot2 package for graphical representation (102).

### MALDI-MSI and data analyses

The liver tissues were subjected to cryopreservation by placing them into a cryochamber at −21 °C for 15 min and subsequently cutting them into longitudinal sections of 20 μm thickness using a cryotome (HM525 cryostat; Thermo Fisher Scientific, Bremen, Germany). These sections were then freeze-mounted onto glass slides and desiccated in a desiccator for 30 min to prevent water precipitation. Digital light microscopic images of the sections were captured (VHX-5000; Keyence, Osaka, Japan) prior to matrix application. For MALDI-MSI, a high-resolution matrix preparation system (SMALDIPrep; TransMIT GmbH, Giessen, Germany) facilitated the uniform deposition of 70 μL of freshly prepared DHB matrix solution (30 mg/mL in 50:50 acetone/water, 0.1% TFA) for measurements in positive-ion mode, and 120 µL of a solution of 9-aminoacridine (7 mg/mL in 70:30 ethanol/water) for negative-ion mode. All MSI experiments were conducted using a high-resolution atmospheric-pressure autofocusing imaging ion source (AP-SMALDI^5^ AF; TransMIT GmbH) coupled to an orbital trapping mass spectrometer (Thermo Scientific Q Exactive HF, Thermo Fisher Scientific (Bremen) GmbH, Bremen, Germany). The tissue sections were scanned with step sizes of 5 μm. The mass spectrometer operated in both, positive- and negative-ion mode, with a mass range set to *m/z* 300–1,200 and a mass resolution setting of 240,000 at *m/z* 200. The MSI raw data underwent processing using the imaging software package MIRION (version 3.4.64.5, TransMIT GmbH), configured with a histogram bin width of 0.004 atomic mass units (u) (103). Simultaneously, the MSiReader software (v1.1) was utilized (104). Molecular annotations were achieved through the METASPACE platform (105).

## Supplementary Material

pgae104_Supplementary_Data

## Data Availability

The mass spectrometry proteomics data were deposited to the ProteomeXchange Consortium via the PRIDE partner repository (106) with the dataset identifier PXD046814.
