# Supplementary material for: Schistosoma mansoni infection induces hepatic metallothionein and S100 protein expression alongside metabolic dysfunction in hamsters
Source: PNAS Nexus. 2024 Mar 7;3(4):pgae104. doi: 10.1093/pnasnexus/pgae104 (PMC10983833; doi:10.1093/pnasnexus/pgae104)
Supplement: pgae104_Supplementary_Data [file pgae104_supplementary_data.zip › PNASNEXUS-PNASNEXUS-2023-01183R-s11.docx]

**Supplementary Information for**

*Schistosoma mansoni* infection induces hepatic Metallothionein and S100 protein expression alongside metabolic dysfunction in hamsters

Parviz Ghezellou^a^ ⃰, Verena von Bülow^b^, David Luh^a^, Elisa Badin^a^, Wendell Albuquerque^c^,
Martin Roderfeld^b^, Elke Roeb^b^, Christoph G. Grevelding^d^, Bernhard Spengler^a^ ⃰

^a^ Institute of Inorganic and Analytical Chemistry, Justus Liebig University Giessen, 35392 Giessen, Germany

^b^ Department of Gastroenterology, Justus Liebig University Giessen, 35392 Giessen, Germany

^c^ Institute of Food Chemistry and Food Biotechnology, Justus Liebig University Giessen, Heinrich-Buff-Ring 17, 35392 Giessen, Germany

^d^ Institute of Parasitology, Biomedical Research Center Seltersberg (BFS), Justus Liebig University Giessen, 35392 Giessen, Germany

* To whom correspondence should be addressed: Emails: [parviz.ghezellou@anorg.chemie.uni-giessen.de](mailto:parviz.ghezellou@anorg.chemie.uni-giessen.de); [Bernhard.spengler@anorg.chemie.uni-giessen.de](mailto:Bernhard.spengler@anorg.chemie.uni-giessen.de)

**This PDF file includes:**

Figures S1. H&E liver histology sections of non- (ni), single-sex- (ss), and bisex- (bs) infected hamsters.

Figure S2. Number of identified protein groups and unique peptides.

Figure S3. Hierarchical clustering between sets of proteomic experimental replicates.

Figure S4. immunohistochemical (IHC) staining of hepatic MT2 and S100a6 proteins in the liver tissues.

Figure S5. LC-MS/MS lipidomics of livers of bisex- and non-infected hamsters.

Figure S6. Lipid pathway analysis of bs-infected hamsters.

Figure S7. MALDI-MSI images of lyso-phospholipid species in the livers of bs-infected hamsters.

Figure S8. LipidSearch software processing parameters.

Figure S9. Assessing the quality and reproducibility of lipidomic data.

Tables S1 to S9. Supplementary Tables are provided in an Excel file accompanying this manuscript.


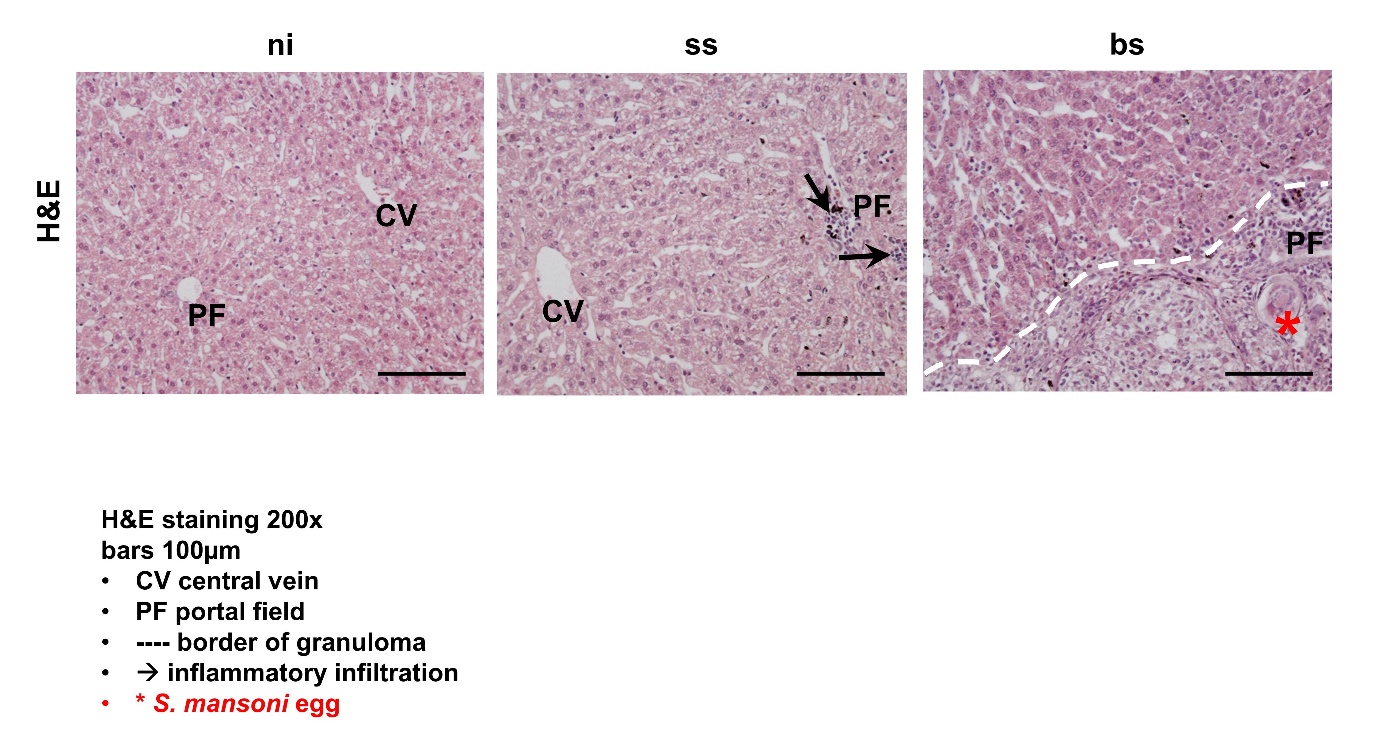


**Figure S1.** Representative liver histology sections of non- (ni), single-sex- (ss), and bisex- (bs) infected hamsters (stained with H&E). CV: central vein, PF: portal field, dash line: border of granuloma, arrows: inflammatory infiltration, and asterisk: *S. mansoni* egg. Magnification 200x, scale bars 100µm.

| Identification | Without fractionation | With fractionation |
| --- | --- | --- |
| #proteins | **1,876** | **4,253** |
| #unique peptides | **17,433** | **25,899** |

**Figure S2.** Number of identified protein groups and unique peptides using pre-fractionation (high-pH reversed phase columns) and non-fractionated samples. The data obtained by using MaxQuant software for data analysis.


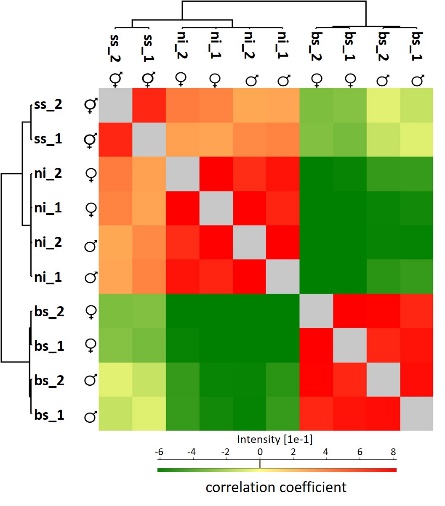


**Figure S3.** The hierarchical clustering graph effectively demonstrated the substantial similarity and reproducibility among proteomic experimental replicates, encompassing bisex (bs), single-sex (ss), and non-infected (ni) male and female hamsters. The clustering analysis unveiled a pronounced divergence between the hepatic proteomes of infected (bs) and non-infected (ni) hamsters. To quantify the proximity of experimental sets, the Pearson correlation coefficient was employed within Perseus software, ensuring the reliability of comparisons and highlighting the consistency of protein abundance patterns across diverse experimental conditions.


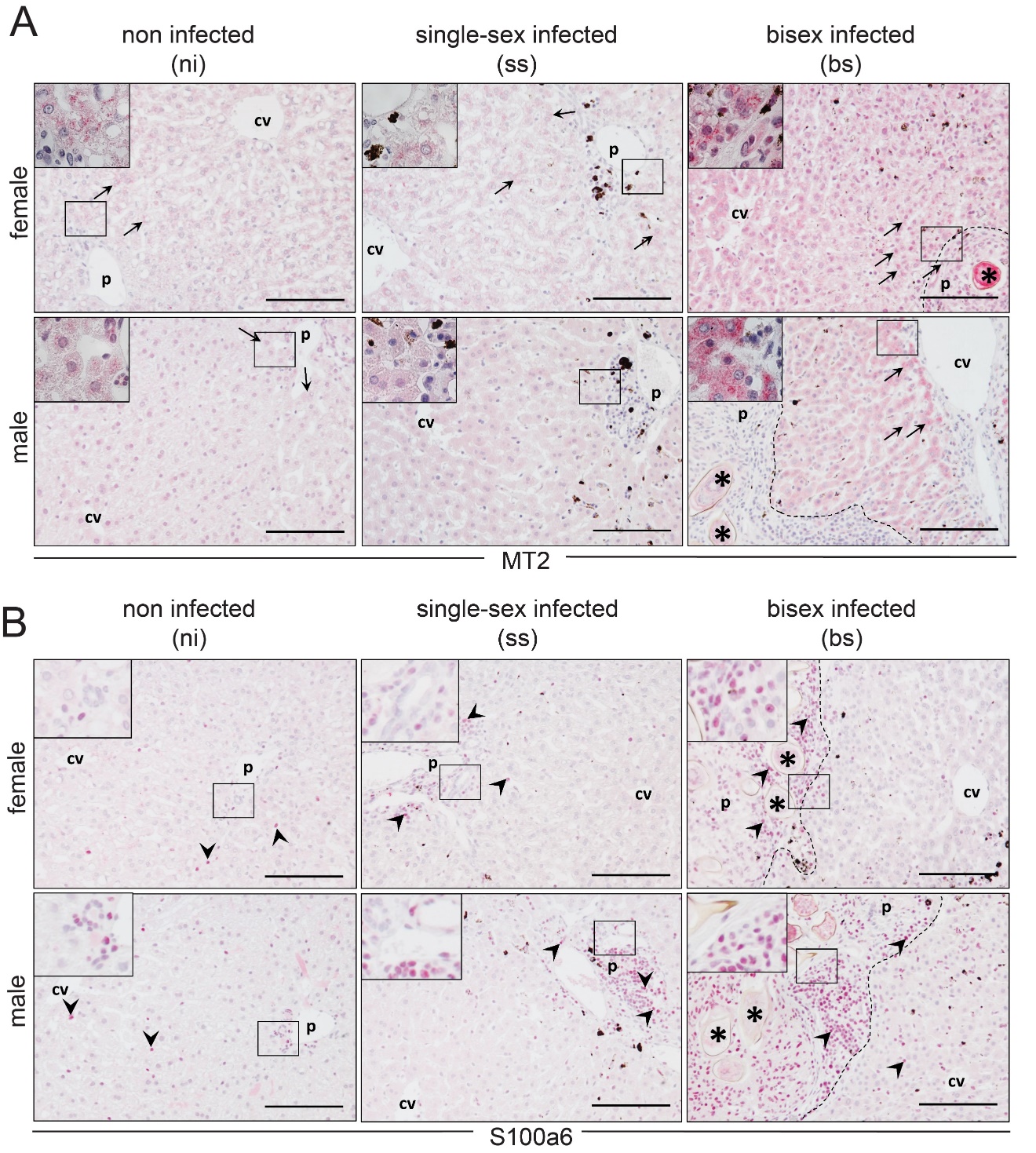


**Figure S4.** A, immunohistochemical (IHC) staining of hepatic MT2 protein in the liver tissues. B, IHC staining of hepatic S100a6 protein in the liver tissues. CV: central vein, PF: portal field, dash line: border of granuloma, arrows: inflammatory infiltration, asterisk: *S. mansoni* egg, and scale bar: 100 µm.


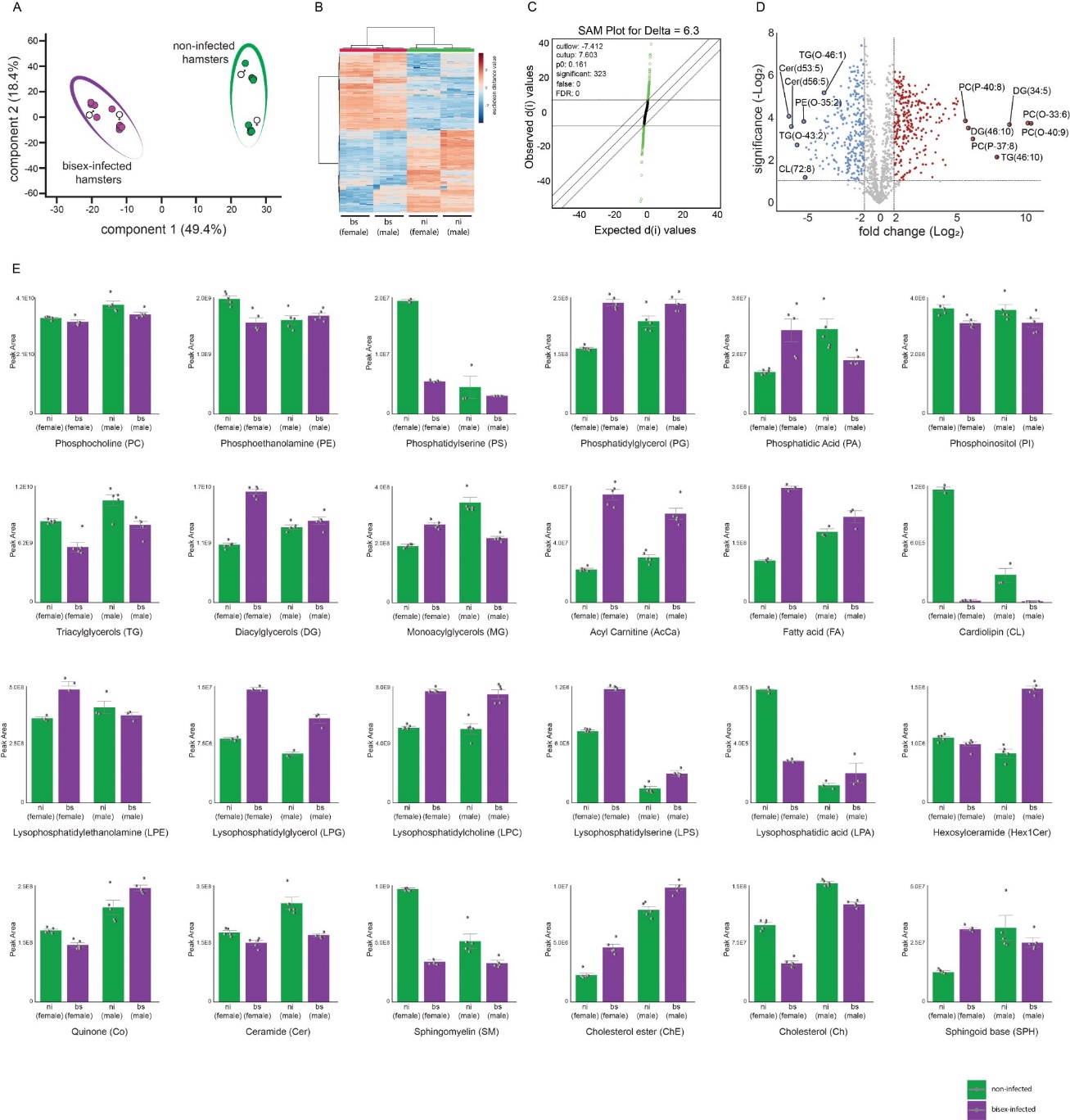


**Figure S5.** LC-MS/MS lipidomics of livers of bisex- and non-infected hamsters. A, principal component analysis (PCA) across all samples with five technical replicates revealed distinctly separated groups of liver lipidome data between bisex- (bs) and non-infected (ni) hamsters. This suggests that the infection status (bs) has a significant impact on the liver lipidome. B, heatmaps of lipid features based on LC-MS/MS lipidomics data of liver samples grouped as female and male hamsters, bisex-infected (bs), and non-infected (ni), in positive-ion mode. The colors in the heat map indicate the log-transformed values of each metabolite. C, significant analysis of metabolites (SAM) plot. The green dots represent lipid features that are differentially regulated between bs and ni hamsters (delta value= 6.3 and false discovery rate = 0). D, volcano plots depict the fold change (Log_2_) in relation to significance (-Log10) of hepatic lipidome for bs relative to control (ni) with combining female and male data; up-regulated (red), down-regulated (blue), and unchanged (gray) expressed proteins. E, the bar charts represent relative quantitation of each lipid class. Each bar is color-coded to differentiate between control and infected samples (male and female) for easy visual comparison.


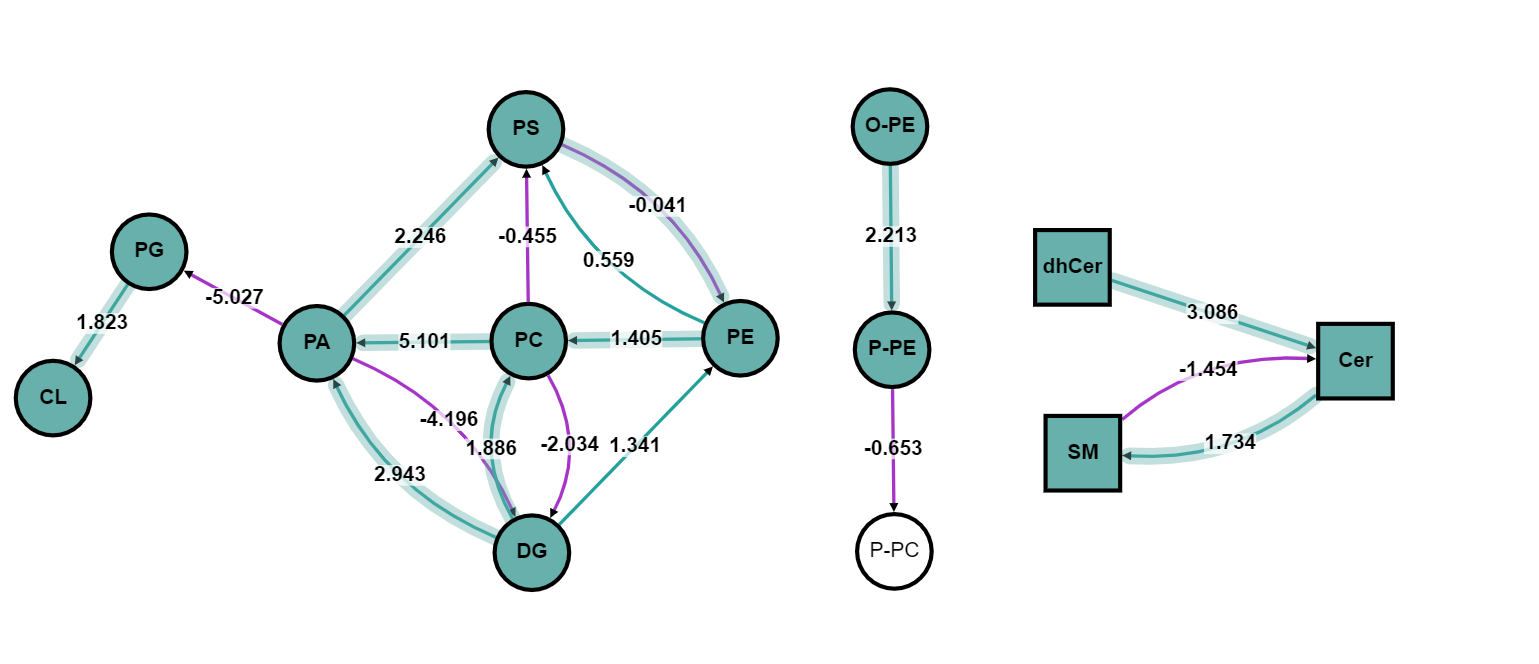


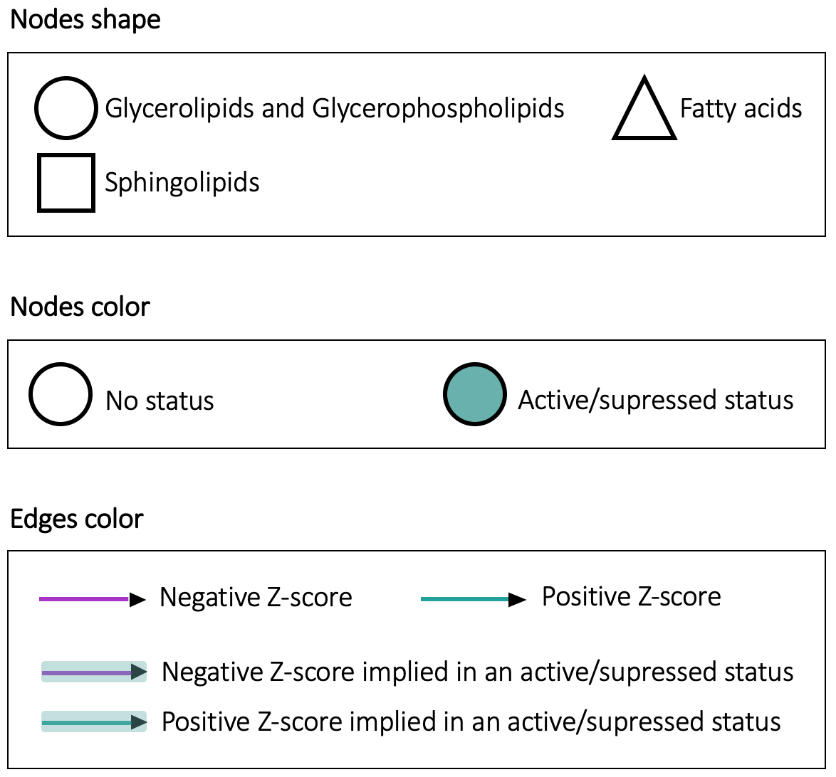


**Figure S6.** A comparison of the lipid pathway activity in bs-infected hamsters with non-infected ones. The lipid classes with the most suppressed reactions and pathways are indicated by green circles. The major anticipated lipid flux across the network is indicated by green arrows. CL: cardiolipin, PA: phosphatidic acid, PI: phosphatidylinositol, Cer: ceramide, PG: phosphatidylglycerol, TG: triacylglycerol, SM: sphingomyelin, PC: phosphatidylcholine, DG: diacylglycerol, dhCer: dihydroceramides, P-: plasmalogen, O-: ether.


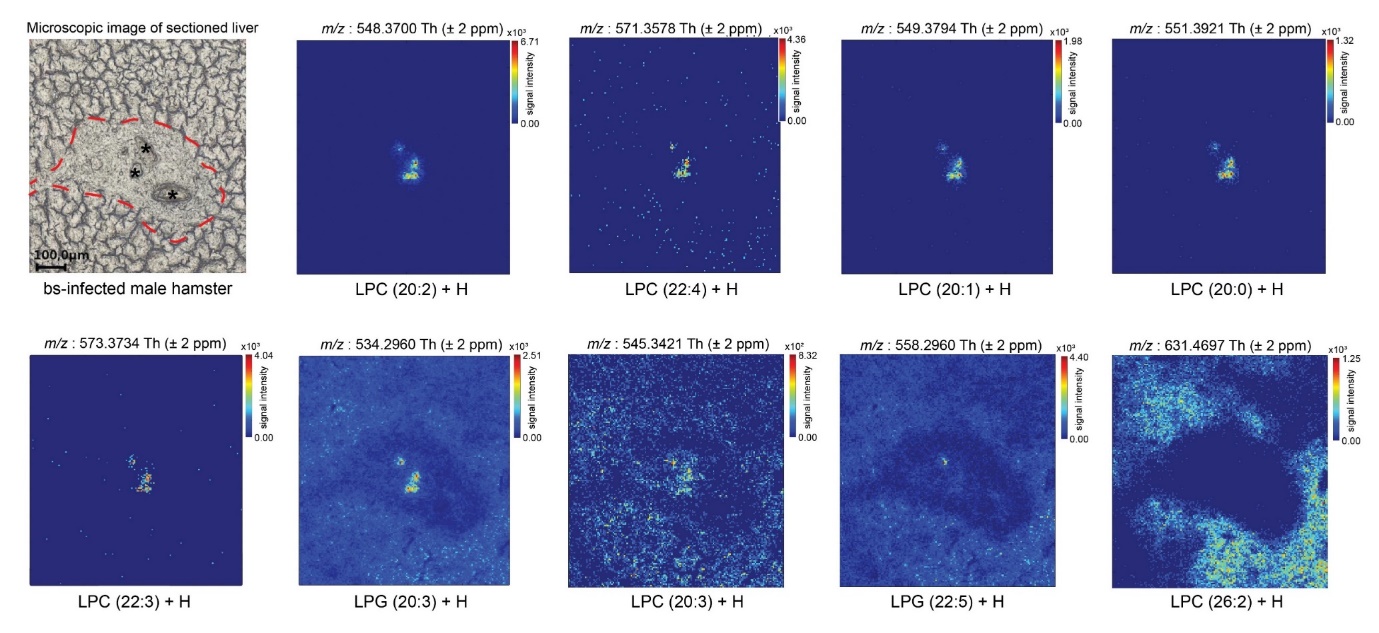


**Figure S7.** MALDI-MSI analyses visualize the distribution of lysolipid species in the liver sections of bs-infected hamsters. LPC: lyso-phosphatidylcholine, LPG: lyso-phosphatidylglycerol, red dash line: border of granuloma, and asterisk: *S. mansoni* egg.

| Parameter | setting |
| --- | --- |
| Process database | |
| Fatty acyls | AcCa |
| Glycerolipids | DG, MG, TG |
| Phospholipids | LPA, PA, LPC, PC, LPE, PE, LPG, PG, LPI, PI, LPS, PS |
| Prenol lipids | Co |
| Sphingolipids | Cer, Hex1Cer, Hex2Cer, Hex3Cer, SM, SPH |
| Sterol lipids | Sterol lipids Ch, ChE, d7-ChE, CmE |
| Identification | |
| Precursor tolerance (ppm) | 3.0 |
| Product tolerance (ppm) | 5.0 |
| Product ion abundance, % | 1.0 |
| Match-score minimum | 5.0 |
| Alignment | |
| Retention time tolerance (min) | 0.1 |
| Data point threshold | 5 |
| Intensity ratio threshold | 3.0 |
| Signal-to-noise ratio | 5.0 |
| Area coefficient of variation, % | ≤30 |

**Figure S8.** LipidSearch software processing parameters, selected lipid classes, and filters


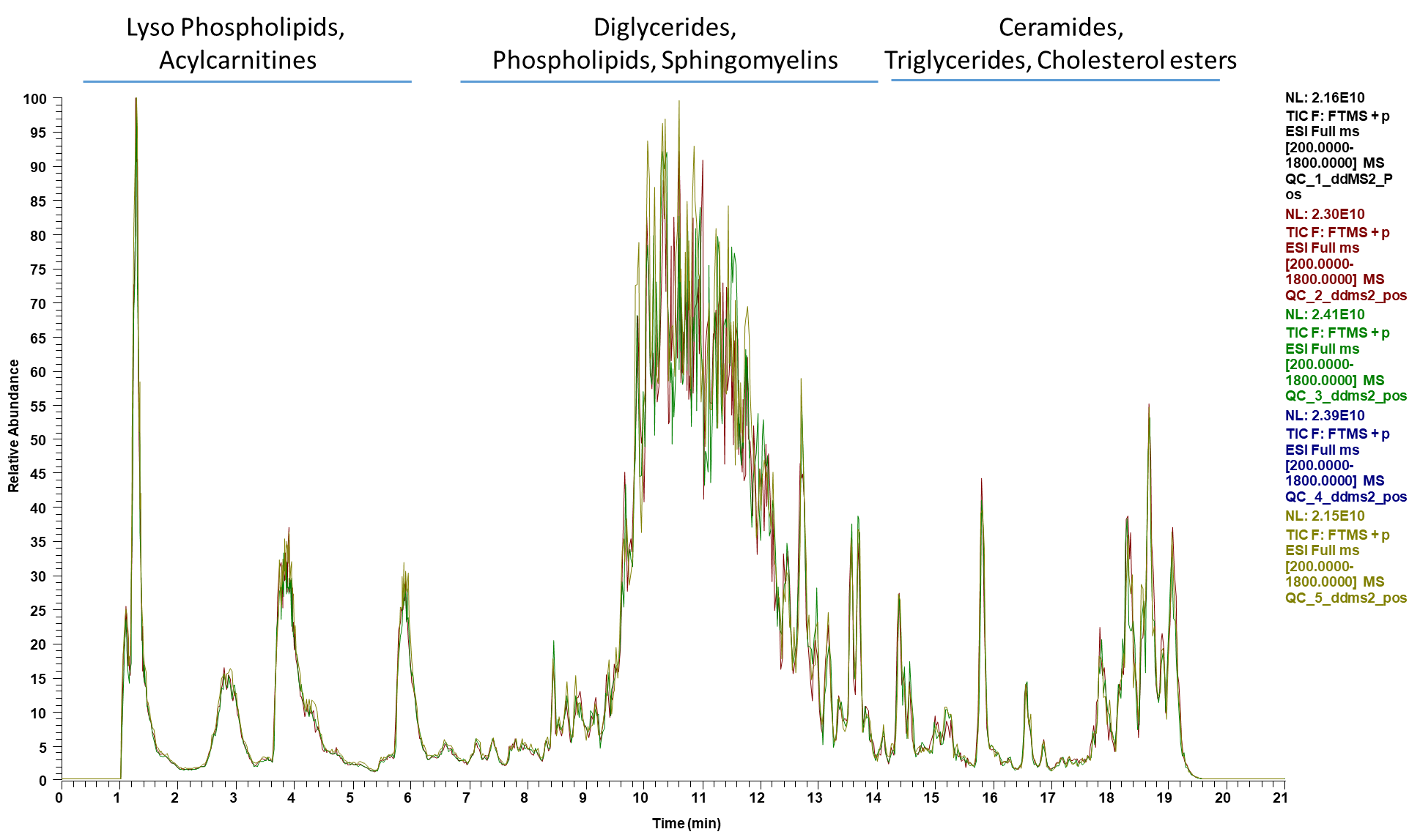


**Figure S9.** Reproducibility of base peak chromatograms from pooled hamster liver extracts as a QC with five replicates. Overlay of positive-ion ESI base peak intensity chromatograms (*m/z* 200-1800) from five replicates of the pooled liver extract (QC) showing the retention time for various lipid sub-classes.

**Tables**

**Tables S1.** Information on the all identified protein groups (4253 protein groups) using high-pH reversed-phase peptide fractionation of TMT-labelled sample before nLC-MS/MS.

**Table S2**. Information on the all identified protein group (1876 protein groups) using TMT-labelled sample without fractionation before nLC-MS/MS.

**Table S3.** List of all hepatic regulated proteins (3,254 protein groups) by comparing all categorized hamsters after filtering based on valid value (min 70%) in Perseus software.

**Table S4.** List of hepatic regulated proteins (3,254 protein groups) by comparing bisex- (bs) versus non-infected (ni) male hamsters.

**Table S5.** List of hepatic regulated proteins (3,254 protein groups) by comparing bisex- (bs) versus non-infected (ni) female hamsters.

**Table S6.** List of regulated proteins (3,254 protein groups) by comparing single-sex- (ss) versus non-infected (ni) male and female hamsters.

**Table S7.** Downregulated proteins involved in the energy metabolic pathways.

**Table S8.** Downregulated proteins involved in the main metabolic pathways.

**Table S9.** Output dataset of LC-MS/MS relative quantitative lipidomics.
